# von Willebrand factor contributes to poor outcome in a mouse model of intracerebral haemorrhage

**DOI:** 10.1038/srep35901

**Published:** 2016-10-26

**Authors:** Ximin Zhu, Yongliang Cao, Lixiang Wei, Ping Cai, Haochen Xu, Haiyu Luo, Xiaofei Bai, Lu Lu, Jian-Ren Liu, Wenying Fan, Bing-Qiao Zhao

**Affiliations:** 1 State Key Laboratory of Medical Neurobiology, Collaborative Innovation Center for Brain Science, Shanghai Medical College and Institutes of Brain Science, Fudan University, Shanghai 200032, China; 2 Department of Neurology, Shanghai Ninth People’s Hospital, Shanghai Jiao Tong University School of Medicine, Shanghai 200011, China

## Abstract

Spontaneous intracerebral haemorrhage (ICH) is the most devastating stroke subtype and *has no proven treatment.* von Willebrand factor (VWF) has recently been demonstrated to promote inflammation processes. The present study investigated the pathophysiological role of VWF after experimental ICH. Functional outcomes, brain edema, blood-brain barrier (BBB) permeability, cerebral inflammation and levels of intercellular adhesion molecule-1 (ICAM-1) and matrix metalloproteinase-9 (MMP-9) were measured in a mouse model of ICH induced by autologous blood injection. We show that VWF were increased in the plasma and was accumulated in the perihematomal regions of mice subjected to ICH. Injection of VWF resulted in incerased expression of proinflammatory mediators and activation of ICAM-1 and MMP-9, associated with elevated myeloperoxidase, recruitment of neutrophils and microglia. Moreover, mice treated with VWF showed dramatically decreased pericyte coverage, more severe BBB damage and edema formation, and neuronal injury was increased compared with controls. In contrast, blocking antibodies against VWF reduced BBB damage and edema formation and improved neurological function. Together, these data identify a critical role for VWF in cerebral inflammation and BBB damage after ICH. The therapeutic interventions targeting VWF may be a novel strategy to reduce ICH-related injury.

von Willebrand factor (VWF) is an ultra-large multimeric glycoprotein, which is present in Weibel-Palade bodies of endothelial cells and alpha granules of platelets and is released in circulation upon activation^1^. VWF plays a crucial role in platelet adhesion and aggregation after vascular injury and under conditions of high shear stress^2^,^3^. Recently, VWF was also shown to mediate leukocyte extravasation and inflammatory response. Animal studies have shown that VWF deficiency reduced inflammatory cell recruitment, atherosclerotic lesion and ischemic cerebral injury^4^,^5^,^6^, and blocking antibody against VWF inhibited neutrophil extravasation in peritonitis and was protected from myocardial ischemic injury^5^,^7^.

Spontaneous intracerebral haemorrhage (ICH), defined as spontaneous bleeding from intraparenchymal blood vessels in the absence of trauma, represents roughly 10–15% of all stroke subtypes^8^. ICH actives immune cells and increases release of inflammatory mediators. As a result, immune cells infiltrates into the brain parenchyma and enhances disruption of the blood-brain barrier (BBB) and the resultant perihematomal edema formation and brain injury^9^. Thus, ICH-induced inflammation is an important factor affecting brain injury, which suggests that anti-inflammatory approaches may lessen the outcome of ICH.

It remains to be elucidated whether VWF also has a pathophysiological role after ICH. Therefore, in the present study we examined the hypothesis that VWF may exert its effect on inflammatory response, BBB dysfunction and associated brain injury after ICH.

## Results

### VWF was induced by ICH injury

**Figure 1:**
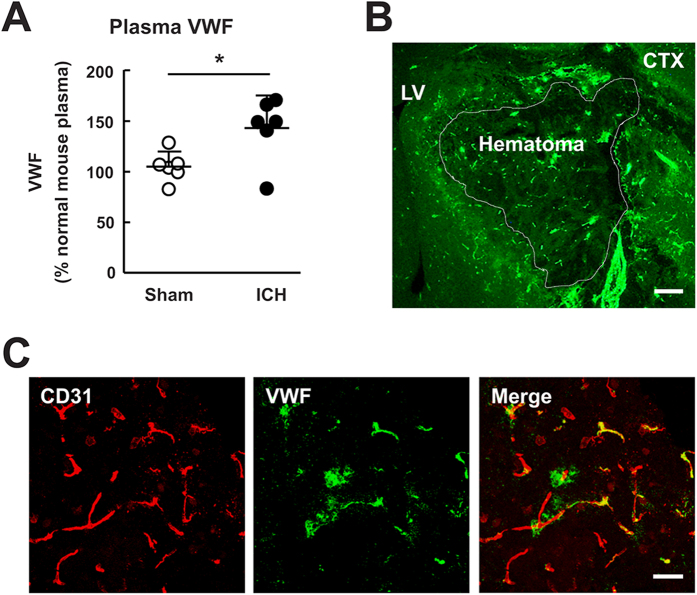
Release of VWF after autologous blood induced intracerebral haemorrhage. (**A**) Mice were subjected to either intracerebral hemorrhage (ICH) or sham surgery. At 24 hours after surgery, plasma samples were taken and analyzed for VWF antigen levels. Values are means ± standard errors of the means (n = 6), *P < 0.05. (**B)** Representative image of VWF immunostaining. VWF was upregulated in the hemorrhagic regions, especially in the perihematomal regions of the brain. (**C**) Double immunostaining of VWF with endothelial cells (CD31) in the perihematomal area. Bar = 200 μm (**B**) and 20 μm (**C**). LV, lateral ventricle; CTX, cortex.

We examined the expression of VWF in mice subjected to 24 hours of ICH. ELISA analysis showed that ICH resulted in significantly increased plasma VWF levels compared with sham-operated mice (Fig. 1A, *P* < 0.05). Immunostaining showed robust VWF accumulation in the hemorrhagic regions, especially in the perihematomal regions of the brain after ICH (Fig. 1B). Double immunostaining indicated colocalization of VWF with endothelial cells (Fig. 1C). These data suggested that VWF was released from activated endothelial cells after ICH. Future studies will be required to determine if VWF present in plasma will get deposited into the ECM. Furthermore, whether VWF was present in autologous blood remain to be clarified.

### VWF increased BBB permeability and brain water content

**Figure 2:**
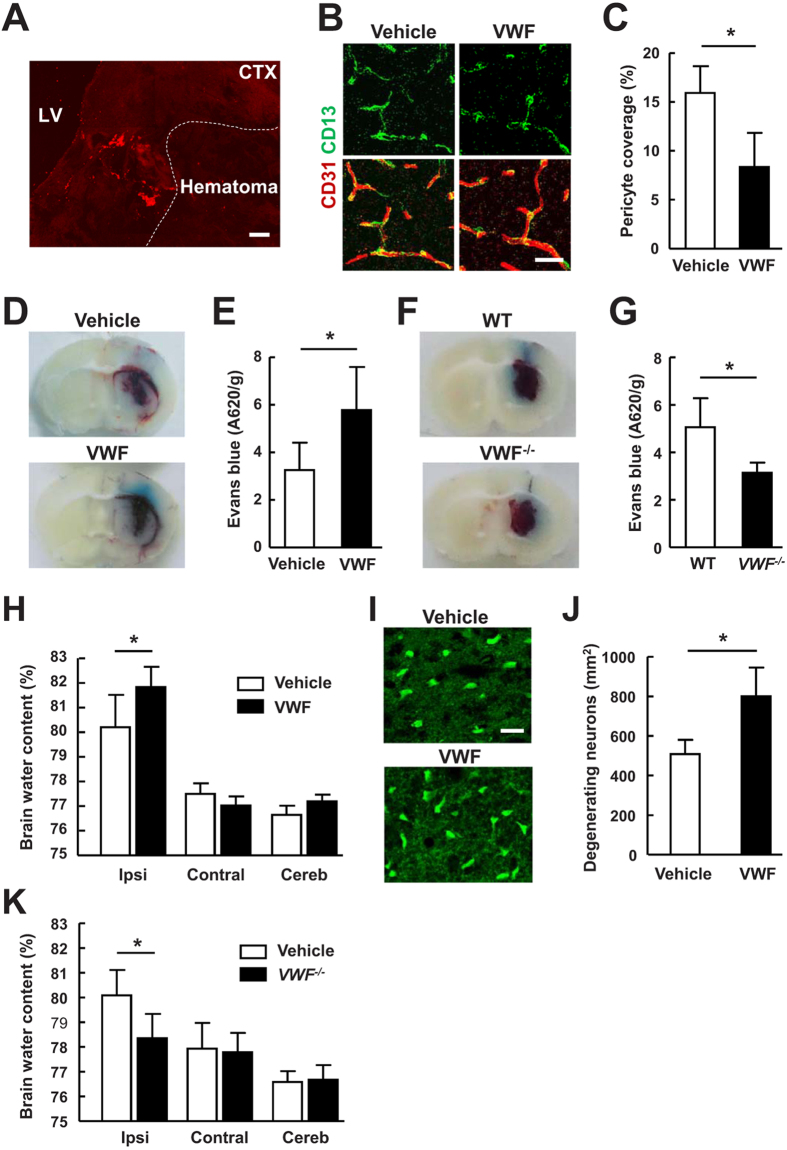
VWF increased BBB damage, brain water content and degenerating neurons after ICH. (**A**) Distribution of the injected VWF protein was tracked by an anti-VVW antibody. LV, lateral ventricle; CTX, cortex. (**B**) Double immunostaining of CD13-positive pericyte (green) with CD31-positive brain endothelium (red) showing pericyte coverage of brain microvessels in the perihematomal area of mice treated with vehicle or VWF 24 hours after ICH. Bar = 30 μm. (**C**) Quantitative analysis of CD13-positive pericyte coverage of CD31-positive brain capillaries in the perihematomal area. Values are means ± standard errors of the means (n = 5). (**D**) Representative photographs of cerebral coronal sections show Evans blue dye extravasation (blue staining) 24 hours after ICH in mice treated with vehicle or VWF. (**E**) Quantification of Evans blue dye extravasation by spectrophotometric assay. Values are means ± standard errors of the means (n = 8). *P < 0.05. (**F**) Representative photographs of cerebral coronal sections show Evans blue dye extravasation 24 hours after collagenase-induced ICH in WT and *VWF*^−/−^ mice. (**G**) Quantification of Evans blue dye extravasation by spectrophotometric assay. Values are means ± standard errors of the means (n = 8). *P < 0.05. (**H**) Brain edema measured 72 hours after ICH in mice treated with vehicle or VWF. Ipsi, ipsilateral hemisphere; Contral, contralateral hemisphere; Cereb, cerebellum. Values are means ± standard errors of the means (n = 8). *P < 0.05. (**I,G**) Representative images of Fluoro-Jade B (FJB) staining (**I**) and quantification of FJB–positive neurons (**J**) in brain sections from mice treated with vehicle or VWF 72 hours after ICH. Bar = 20 μm. Values are means ± standard errors of the means (n = 5). *P < 0.05. (**K**) Brain edema measured 72 hours after collagenase-induced ICH in WT and *VWF*^−/−^ mice. Values are means ± standard errors of the means (n = 8). *P < 0.05.

In order to determine the role of VWF in ICH, the exogenous VWF was used. The distribution of the injected VWF protein was tracked by an anti-VWF antibody that does not bind rodent VWF. VWF staining was observed in intraventricular areas (data not shown) as well as the brain parenchyma (Fig. 2A). Pericytes contribute to the maintenance of BBB integrity^10^. Studies have shown that pericyte loss leads to brain vascular damage^11^. Recently, Hall *et al*. reported that ischemia-induced pericyte constriction and death may cause BBB breakdown^12^. Subsequent studies using animal models of cerebral ischemia also demonstrated a link between pericyte loss and BBB disruption^13^. Using the *pericyte* marker, CD13^14^,^15^, we quantified pericyte coverage in the perihematomal areas at 24 hours after ICH. Treatment with VWF resulted in a marked decrease in pericyte coverage (Fig. 2B,C, *P* < 0.05) compared with vehicle-treated mice. We also measured the BBB permeability by Evans blue extravasation. In mice treated with VWF, the extravasation of Evans blue dye was increased by 79% in the ipsilateral hemisphere (Fig. 2D,E, *P* < 0.05). Brain water content in ipsilateral hemispheres was 80.21 ± 1.31% in the vehicle-treated group, and 81.84 ± 0.83% in the VWF-treated group (Fig. 2H, *P* < 0.05), whereas in contralateral hemispheres these were 77.50 ± 0.43% in the vehicle-treated group and 77.03 ± 1.05% in the VWF-treated group (*P* > 0.05), showing that VWF significantly exacerbated brain edema in hemorrhagic hemispheres. In a collagenase-induced ICH model, we further demonstrated that *VWF*^−/−^ mice exhibited significantly reduced BBB permeability and brain water content compared with wild-type mice (Fig. 2F,G,K). The findings are consistent with a recent study showing that VWF deficiency decreased BBB permeability in mouse models of hypoxia/reoxygenation and pilocarpine-induced status epilepticus^16^. The same authors showed that there was no significant differences in BBB permeability at baseline between wild type and *VWF*^−/−^ mice. Furthermore, VWF deficiency in mice decreases neutrophil recruitment and anti-VWF antibodies inhibited permeability in peripheral tissues^7^,^17^,^18^. The difference between the present findings and a previous report suggesting that VWF deficiency in mice increases BBB permeability in experimental allergic encephalomyelitis could be explained by the different disease models used^19^. In addition, Fluoro-Jade B stained neurons (Fig. 2I,J, *P* < 0.05) were significantly increased in the lesioned hemispheres by VWF treatment.

### VWF exacerbated cerebral inflammation after ICH

**Figure 3:**
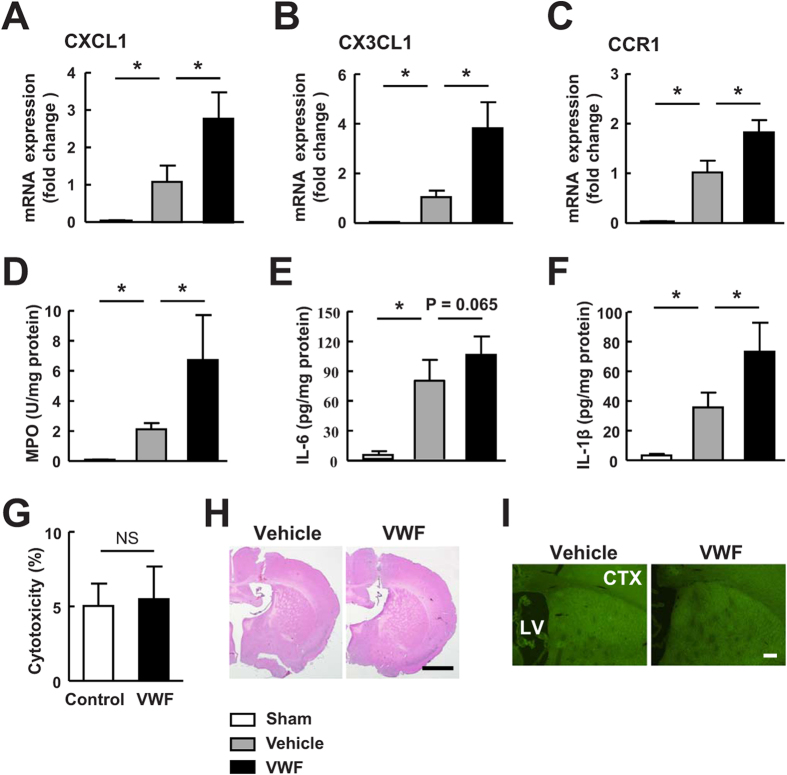
VWF increased the expressions of cerebral inflammatory mediators after ICH. (**A–C**) Relative gene expression of CXCL1, CX3CL1 and CCR1 in the brain of sham-operated mice and mice treated with vehicle or VWF 24 hours after ICH. (**D–F**) Quantification of MPO activity, IL-6 and IL-1β by ELISA in the brain of sham-operated mice and mice treated with vehicle or VWF 24 hours after ICH. Values are means ± standard errors of the means (n = 5). *P < 0.05. (**G**) LDH assay confirmed that the treatment of VWF did not induce cell death in cultured brain endothelial cells. Data are representative of 3 independent experiments. (**H**) Intraventricular injection of VWF did not show histological features of neuronal injury at 72 hours when examined with hematoxylin and eosin staining. (**I**) Intraventricular injection of VWF did not induce neurotoxic in normal mice at 72 hours when detected with TUNEL (terminal deoxynucleotidyl transferase-mediated dUTP nick-end labeling) staining. LV, lateral ventricle; CTX, cortex.

**Figure 4:**
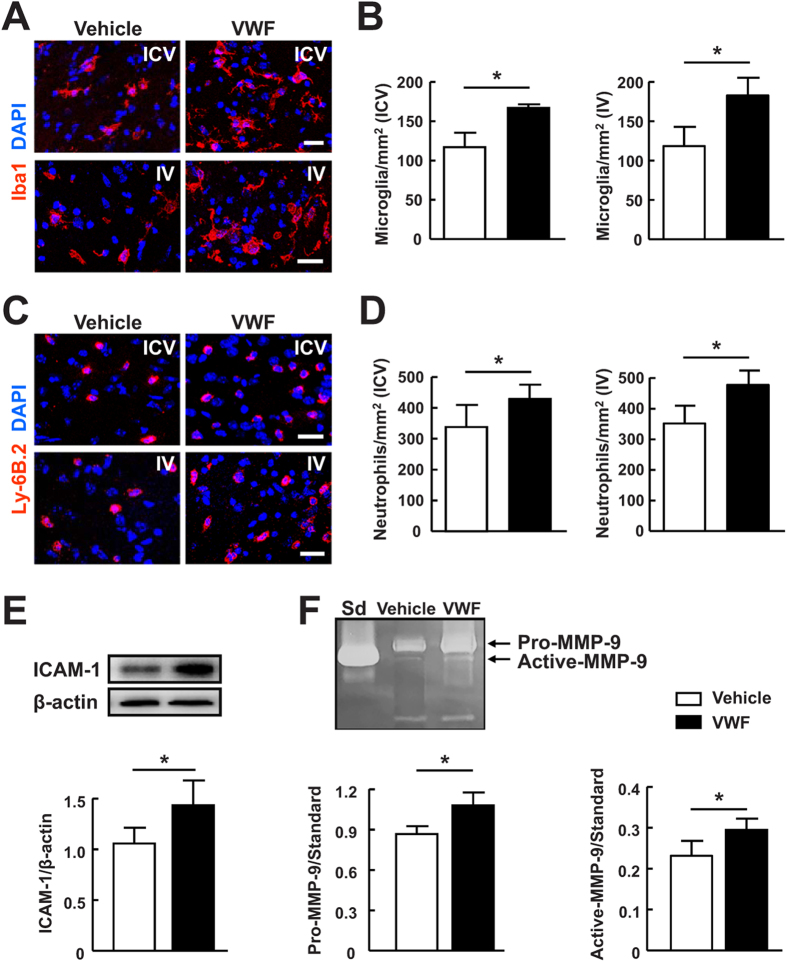
VWF increased inflammatory cell recruitment, ICAM-1 and MMP-9 levels after ICH. (**A,C**) Representative images of microglia and neutrophils immunostaining in the perihematomal area 24 hours after ICH in mice treated with either intraventricular (ICV) or intravenous (IV) injection of VWF. Nuclei were visualized with DAPI. Bar = 15 μm. (**B,D**) Quantitative analysis of activated microglia and neutrophils in the perihematomal area. Values are means ± standard errors of the means (n = 5). *P < 0.05. Microglia were considered activated when the cell body exhibited stout morphology with retracted processes. (**E**) Representative immunoblots and quantification of ICAM-1 in the brain extracts from mice treated with vehicle or VWF 24 hours after ICH. Values are means ± standard errors of the means (n = 5). (**F**) Representative MMP-9 gelatin zymography and quantitative analysis of pro-MMP-9 and active-MMP-9 in the brain extracts from mice treated with vehicle or VWF 24 hours after ICH. Sd, Human MMP-9. Values are means ± standard errors of the means (n = 5). *P < 0.05.

We analyzed the gene expression profiles of several chemokines in the hemorrhagic brains 24 hours after ICH. The results revealed that ICH strongly elevated the levels of chemokine (C-X-C motif) ligand 1 (CXCL1), CX3CL1 and the chemokine (C-C motif) receptor (CCR1) compared with sham-operated group (Fig. 3A–C, *P* < 0.05). Treatment with VWF resulted in even greater increase in these chemokines compared with vehicle-treated mice (*P* < 0.05). Myeloperoxidase (MPO), a local indicator of inflammation^20^, was substantially elevated by ICH (Fig. 3D). We then found a marked increase in MPO activity in mice subjected to ICH and VWF treatment compared to mice treated with ICH and vehicle (*P* < 0.05). Accordingly, we found that the proinflammatory cytokines interleukin-6 (IL-6) and IL-1β were dramatically elevated in the brains of both vehicle-treated and VWF-treated mice 24 hours after ICH compared with sham-operated mice, and the expression was higher in mice treated with VWF (Fig. 3E,F). We also characterized the inflammatory response after ICH by immunohistochemical quantification of the numbers of inflammatory cells in the brains. This analysis showed that both intraventricular and intravenous administration of VWF significantly increased microglial activation (Fig. 4A,B, *P* < 0.05) and neutrophil accumulation (Fig. 4C,D, *P* < 0.05) compared with vehicle-treated mice. Activated microglia and neutrophil extravasation were almost undetectable in the sham group (data not shown). Taken together, these data indicate that VWF contributed to brain inflammation after ICH.

The detrimental roles of intercellular adhesion molecule-1 (ICAM-1) and Matrix metalloproteinase-9 (MMP-9) have been well documented in the literature with regard to their effects on inflammatory responses and BBB integrity following brain injury^21^,^22^,^23^. Our data showed that VWF regulated brain inflammation and BBB permeability after ICH. Therefore, we tested whether VWF could modify ICAM-1 and MMP-9. Western blot analysis revealed that cerebral ischemia significantly increased ICAM-1 expression compared with the sham-operated group (Fig. 4E, *P* < 0.05). Gelatin zymography demonstrated an increase in pro-MMP-9 and activated MMP-9 in VWF-treated mice (Fig. 4F, *P* < 0.05).

### Blocking antibodies against VWF improved functional outcomes, BBB permeability and reduced brain water content after ICH

**Figure 5:**
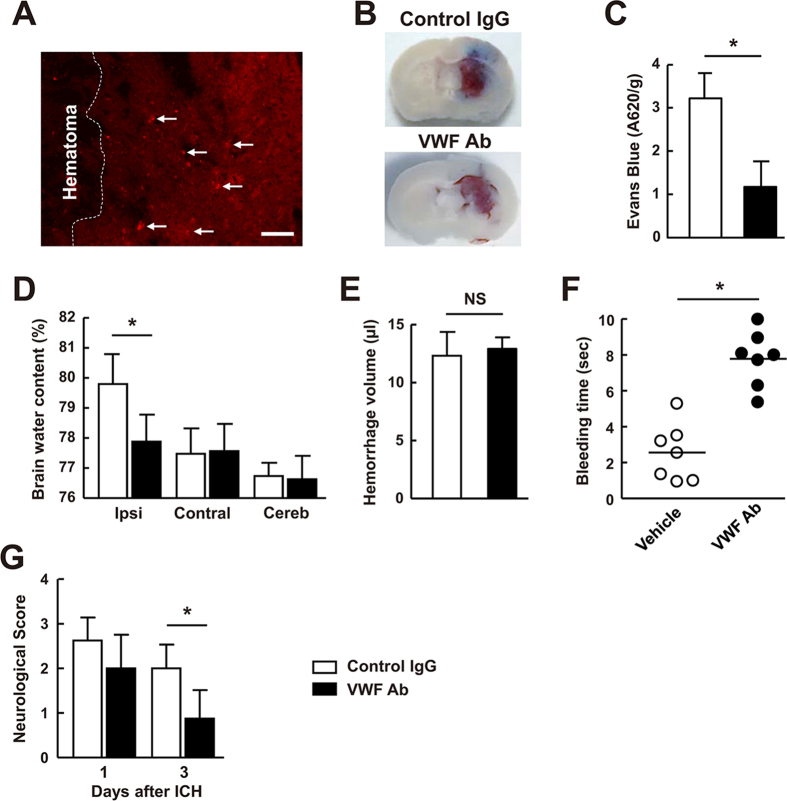
Antibodies against VWF improved neurological functions and reduced brain edema after ICH. (**A**) Distribution of the injected anti-VWF antibody in the brain was labeled with an anti-rabbit secondary antibody. (**B**) Representative photographs of cerebral coronal sections show Evans blue dye extravasation (blue staining) 24 hours after ICH in mice treated with control rabbit IgG or rabbit anti-VWF antibody. (**C**) Quantification of Evans blue dye extravasation by spectrophotometric assay. Values are means ± standard errors of the means (n = 8). *P < 0.05. (**D**) Brain edema measured 72 hours after ICH in mice treated with control rabbit IgG or rabbit anti-VWF antibody. Ipsi, ipsilateral hemisphere; Contral, contralateral hemisphere; Cereb, cerebellum. Values are means ± standard errors of the means (n = 8). *P < 0.05. (**E**) Hemorrhagic volume measured 24 hours after ICH by spectrophotometric hemoglobin assay in mice treated with control rabbit IgG or rabbit anti-VWF antibody. Values are means ± standard errors of the means (n = 5). NS, not significant. (**F**) Tail bleeding time measured 24 hours after ICH in mice treated with control rabbit IgG or rabbit anti-VWF antibody. *P < 0.05. (**G**) Neurological score in mice treated with control rabbit IgG or rabbit anti-VWF antibody 72 hours after ICH. Values are means ± standard errors of the means (n = 8). ^*^P < 0.05.

We next studied the role of VWF inhibition using function blocking antibodies against VWF in mice subjected to ICH. Antibodies against VWF was administered intravenously following ICH. The distribution of the injected anti-VWF antibody in the brain was labeled with an anti-rabbit secondary antibody. Fluorescence of labeled VWF was observed in brain parenchyma (Fig. 5A). All animals survived after infusing anti-VWF antibodies. We found that anti-VWF antibodies significantly reduced BBB permeability and brain water content in the ipsilateral hemispheres compared to the control IgG group (Fig. 5B–D, *P* < 0.05). Anti-VWF antibodies did not change brain water in the contralateral hemisphere. By using a spectrophotometric hemoglobin assay^6^, we found that intravenous injection of anti-VWF antibodies did not affect hemorrhage volume compared to the control IgG group (Fig. 5E), indicating that anti-VWF antibodies had no effect on bleeding in the brain after ICH. Although anti-VWF antibodies prolonged the bleeding time (Fig. 5F), this did not simply reflect the risk of hemorrhage in the brain after ICH in the current study. Our results strongly confirm the previous finding that no direct correlation exists between bleeding time and bleeding risk in the brain^24^. In addition, anti-VWF antibodies-treated mice exhibited less severe neurological deficits as assayed by the neurological scores compared with mice receiving control IgG administration (Fig. 5G). The injection of anti-VWF antibodies at 10 minutes after ICH is early, additional future studies will be required to determine if delayed inhibition of VWF can protect against ICH-induced brain injury.

## Discussion

The major findings of this study are: (1) VWF was upregulated in the plasma and was accumulated in endothelial cells in the perihematomal regions after ICH; (2) VWF exacerbated cerebral inflammation, BBB impairment and neuronal injury after ICH; and (3) Blocking VWF provided a significant protective effect on brain edema and improved functional outcomes after ICH.

Release of local proinflammatory cytokines and the subsequent upregulation of endothelial adhesion molecules are the early events after ICH. These events are followed by the damage of endothelial cells and infiltration of immune cells, which likely contribute to brain injury^25^. It was shown that inflammatory stimuli induced secretion of VWF^18^, leading to the deposition of VWF strings on the vessel wall that mediates adhesion of platelets^26^,^27^. Recent findings reveal that platelets bound to VWF contributed to leukocyte adhesion and transmigration, which results in further amplification and propagation of inflammation^28^. Although it is not known whether VWF plays a role in ICH, raised plasma levels of VWF were observed in patients with ICH and subarachnoidal haemorrhage^29^,^30^. Here, we found that VWF levels were significantly elevated in both the plasma and the brain in mice subjected to ICH compared with controls. Furthermore, treatment with VWF dramatically accelerated cerebral inflammation after ICH. This result is consistent with previous reports that showed decreased neutrophil extravasation in VWF-deficient mice after immune complex–mediated vasculitis, atherosclerotic lesions and myocardial ischemia^5^,^18^,^31^. Both ICAM-1 and MMP-9 have been widely implicated as mediators of inflammation in ICH, subarachnoid haemorrhage and cerebral ischemia^32^,^33^. Previous studies have shown that ICAM-1 and MMP-9 also play important roles in the pathogenesis of BBB breakdown following brain injury^34^,^35^. VWF is an acute phase reactant that is released from activated endothelial cells upon injury such as ICH^36^. Both ICAM-1 and MMP-9 expressions are altered in activated endothelial cells^37^,^38^. It is, therefore, not surprising to have an elevated expression of ICAM-1 and MMP-9 after VWF treatment in ICH. Indeed, we found that VWF treatment significantly increased the ICAM-1 expression and MMP-9 activation, which were associated with significantly increased pericyte loss and more severe BBB damage.

An important goal of ICH management is to prevent edema formation because brain swelling may have a greater influence on poor outcome after ICH. Available evidence suggests that inflammatory response and BBB dysfunction contribute to perihematomal edema formation^39^. In the present study, we showed that mice treated with VWF after induction of ICH had increased brain water content compared with vehicle-treated mice. Importantly, we demonstrated that blocking of VWF by anti-VWF antibodies reduced BBB permeability and protected mice from ICH without inducing excessive bleeding. Our findings underline the important pathophysiologic role of VWF during ICH. However, clinical deterioration after intracerebral haemorrhage is not just because of formation of edema around the haemorrhage, but it is also important haemorrhage progresses.

Inflammatory response and BBB dysfunction play important roles in the edema formation and consequent brain injury after ICH. We report that injection of VWF exacerbated ICH-induced brain inflammatory responses and BBB disruption. Our data also demonstrate that treatment with anti-VWF antibodies perserved BBB integrity and reduced brain edema formation. Our study suggest that targeting VWF-mediated inflammation and BBB disruption could become a promising new approach for specific therapy of ICH.

## Methods

### ICH model and treatment regimens

Male C57BL/6J mice (Shanghai SLAC Laboratory Animal Corporation) age 10 to 12 weeks were used. The VWF-deficient mice used in this study were on a C57BL/6J background and were obtained from the Jackson Laboratory. All procedures were conducted in accordance with the Animal Experimentation Guidelines of Fudan University and approved by the Animal Care and Use Committee of School of Basic Medical Sciences, Fudan University. Animals were anesthetized with 1% to 1.5% isoflurane in 30% oxygen with the use of a face mask. Experimental ICH was induced by the double blood injection method as described^40^. To induce moderately large and reproducible brain edema formation and marked neurological deficits, we used 30 μl of autologous blood^39^. Briefly, a 1 mm-diameter burr hole was drilled on the left side of the skull. Thirty microliters of autologous blood was taken from the tail artery into a capillary tube, which was flushed with heparin before blood withdrawal. A 26-gauge needle was advanced stereotactically into the striatum (coordinates: 0.2 mm anterior, 2.3 mm lateral to the bregma, 2.8 mm ventral). Using a microinfusion pump, 5 μl of blood was injected at a rate of 2 μl/minute. The needle was advanced 0.7 mm further, and additional 25 μl of blood was injected at 2 μl/minute after 7 minutes. After a period of 10 minutes, the needle was slowly removed, the burr hole was sealed with bone wax. Purified plasma-derived human VWF (270 ng in 3 μl PBS; Haematologic Technologies, Essex Junction, VT, USA) or PBS was injected directly into the cerebral lateral ventricle at 30 minutes after ICH induction. Intraventricular injection and intravenous injection are two very different methods of injection^41^. Intraventricular injection results in delivery of materials into the CNS through the cerebrospinal fluid^42^,^43^. Previous studies showed that cerebrovascular permeability was increased by intraventricular injection of tissue plasminogen activator (22.75–585 ng) in mice^44^,^45^. However, intravenous injection a higher dose of tissue plasminogen activator (10 mg/kg, ≈250 μg/mouse) did not affect cerebrovascular permeability^45^. VWF (5 μg/mouse) or PBS was also administrated intravenously into mice at 30 minutes after ICH induction^46^. Rabbit anti-VWF antibody (6 μg/g body weight, clone A0082; Dako) or control rabbit IgG was administered intravenously 10 minutes after ICH. Tail bleeding time was determined as previously described^6^.

To examine whether VWF deficiency affects BBB permeability and brain water content, the collagenase-induced ICH model was used^47^. In brief, mice were placed on a stereotactic frame and 0.5 μl of saline containing 0.025 U collagenase VII (Sigma-Aldrich) was injected into the left striatum at coordinates 0.2 mm anterior and 2.3 mm lateral to bregma, and 3.5 mm ventral to the skull surface. Because the *VWF*^−/−^ mice has a highly prolonged bleeding time^6^, we could not control the autologous blood injection at the same speed. Therefore, the collagenase-induced ICH model was used to examine whether VWF deficiency affects BBB permeability and brain water content.

### Measurements of VWF, IL-6 and IL-1β levels

Blood was collected 24 hours after ICH induction by retro-orbital bleeding into polypropylene tubes containing 3.8% trisodium citrate. Plasma was prepared by centrifugation of the blood and stored at −80 °C until analysis. Microtiter plates were coated with 100 μl/well rabbit anti-human VWF (1:600; A0082, DAKO, Carpinteria, CA, USA) in PBS overnight at 4 °C. Plates were washed 3 times with 0.1% Tween 20 and 0.3% bovine serum albumin (BSA; Sigma-Aldrich, St Louis, MO, USA) in PBS, and plasma samples (1:40 diluted with 3% BSA in PBS) were incubated in the wells for 2 hours at room temperature. After washing, the plates were incubated with 100 μl HRP-conjugated VWF (1:2000 diluted with 3% BSA in PBS; DAKO) for 1 hour at room temperature. After washing, 100 μL tetramethylbenzidine substrate (Sigma-Aldrich) was added to the wells for 15 minutes, and the reaction was stopped with 50 μL 2N HCL. Absorbance was measured at 450 nm on an ELISA reader (Bio-Rad, Hercules, CA, USA). A reference standard was generated using normal pooled plasma from 15 mice. The levels of IL-6 and IL-1β were assayed using ELISA kits (R&D systems) according to the manufacturer’s instructions.

### Brain water content

Mice were euthanized by overdose of chloral hydrate and killed 3 days after ICH. Brains were removed immediately and separated into left and right hemispheres, and cerebellum. Each hemisphere was cut into 4 mm thickness block around the needle track. Brain samples were immediately weighted on an electronic analytical balance (Mettler Toledo, Columbus, OH, USA) to obtain the wet weight. Brain samples were dried at 100 °C for 24 hours to obtain the dry weight. Brain water content was calculated as: (wet weight − dry weight)/wet weight × 100%.

### Cell Culture

Mouse brain endothelial cell line (bEnd.3) (American Type Culture Collection, Manassas, VA) was used. Cells were cultured in Dulbecco’s modified Eagle’s medium (DMEM) (Invitrogen, Camarillo, CA) supplemented with 10% fetal calf serum (Invitrogen) and antibiotics in a humidified cell culture incubator at 37 °C and 5% CO_2_^47^. Cells were incubated with VWF (10 ug/ml) for 24 hours^47^. Cytotoxicity was evaluated by a standard measurement of lactate dehydrogenase (LDH) release using the LDH assay kit (repeated three times in triplicate, Roche, Indianapolis, IN).

### Fluoro-Jade B staining

Fluoro-Jade B Staining was performed as described previously^48^. Cerebral tissue cryosections (20 μm) were dehydrated in 100 and 70% ethanol and incubated with 0.06% potassium permanganate for 10 minutes. The sections were then rinsed in distilled water and incubated in 0.0004% Fluoro-Jade B solution (Millipore) in 0.1% acetic acid, rinsed in distilled water, dehydrated, and cleared in xylene. Staining was visualized using an Olympus FV 1,000 confocal microscope. Fluoro-Jade B positive cells were counted in four fields of the perihematoma area at 40 × magnification.

### Real-time PCR

Total RNA was extracted by homogenization of the ipsilateral hemispheres in TRIzol reagent (Invitrogen, Camarillo, CA, USA) and was quantified with spectrophotometry. The RNA was reverse transcribed using RevertAid First Strand cDNA Synthesis Kit (*Fermentas*, Glen Burnie, MD, USA). The PCR was performed using an ABI 7300 PCR machine (Applied Biosystems, Foster City, USA). Gene expression was normalized to β-actin. Primer sequences for CXCL1, CX3CL1, CCR1 and GAPDH were as follows: CXCL1 (forward, 5′-ACTGCACCCAAACCGAAGTC-3′, reverse, 5′-CAAGGGA GCTTCAGGGT-CAA-3′), CX3CL1 (forward, 5′-GCACAGGATGCAGGG CTTAC-3′, reverse, 5′-TGTCAGCCGCCTCAAA-ACT-3′), CCR1 (forward, 5′-CTGAGGGCCCGAA CTGTTAC-3′, reverse, 5′-GGCTAGGGCCCAGGTGAT-3′), GAPDH (forward, 5′-AATGT GTCCGTCGTGGATCTGA-3′, reverse, 5′-GATGCCTGCTTCACCACCTTCT-3′).

### MPO activity

Ipsilateral brain tissues were homogenized in 500 μL of 50 mM potassium phosphate buffer (pH 7.0). The samples were centrifuged, resuspended and subjected to three freeze-thaw cycles in 1% cetyltrimethylammonium bromide (Sigma-Aldrich) in potassium phosphate buffer. Forty microliters of supernatant was added to 100 μL tetramethylbenzidine solution (Sigma-Aldrich), and the optical density was measured at 450 nm after stopping the reaction with 2N HCl. MPO activity was expressed in equivalent units by comparison with a reference curve generated using purified MPO (Sigma-Aldrich).

### Gelatin zymography

Zymography was performed as described^47^. In brief, 40 μg protein samples were mixed with SDS sample buffer and loaded onto 10% Tris-Glycine gels containing 0.1% gelatin. After electrophoresis, gels were washed with 2.5% Triton X-100 for 30 minutes and incubated in developing buffer (50 mM Tris pH 7.6, 0.2 mM NaCl, 5 mM CaCl_2_, 0.02% Brij-35) at 37 °C for 48 hours. Gels were stained with 0.5% Coomassie blue R-250 (Sigma-Aldrich) for 3 hour and destained appropriately. Gels were scanned and analyzed using Quantity One software (Bio-Rad).

### Immunofluorescence

Twenty four hours after ICH induction, mice were euthanized by overdose of chloral hydrate, perfused transcardially with ice-cold PBS and 4% paraformaldehyde (pH 7.4; Sigma-Aldrich). The brains were removed and fixed in 4% paraformaldehyde overnight and then cryoprotected in 30% sucrose overnight at 4 °C. Frozen sections were cut in the coronal plane at 20 μm on a cryostat (Leica Microsystems Inc., Buffalo Grove, IL, USA). Immunohistochemistry was performed as described^49^,^50^. Images were obtained using an Olympus BX 51 microscope and an Olympus FV 1,000 confocal microscope. Colocalization was verified and reconstructed using Olympus FV 10-ASW software. Primary antibodies used were: rabbit anti-VVW (ab154193, Abcam), rabbit anti-human VWF (Dako), rat anti-CD31 (PECAM-1, BD Pharmingen, San Diego, CA, USA), rat anti-mouse LY-6B.2 (AbD Serotec, Raleigh, NC, USA), goat anti-Iba1 (Ionized calcium binding adaptor molecule 1, Abcam, Cambridge, MA, USA) and FITC-conjugated rat anti-mouse aminopeptidase N (CD13) (BD Pharmingen). Secondary antibodies used were: Alexa Fluor 488 donkey anti-rabbit immunoglobulin G (IgG), Alexa Fluor 594 donkey anti-rat IgG, Alexa Fluor 594 donkey anti-goat IgG, and Alexa Fluor 594 donkey anti-rabbit IgG (Invitrogen). Nuclei were stained with DAPI. For quantification of LY6B and Iba1 labeled cells in the perihematoma area, four fields from each section were captured under 40X objective. Each image was traced using ImageJ software. The total numbers of positive cells in the traced area were counted and expressed as per mm^2^. Pericyte coverage was determined as a percentage of CD13-positive area covering CD31-positive area in 0.42 mm^2^ regions. For each animal, 3 sections (400 μm apart) from the ipsilateral hemispheres were analyzed.

### Western blotting

Twenty four hours after ICH induction, mice were transcardially perfused and the brains were removed and separated into hemispheres ipsilateral and contralateral to haemorrhage. Ipsilateral hemispheres were cut into 4 mm thickness block around the needle track. The brain tissues were homogenized in RIPA lysis buffer (Millipore) containing protease inhibitor cocktails (Roche Diagnostics GmbH, Mannheim, Germany). Equal amounts of protein samples were loaded on 10% SDS-PAGE gel, electrophoresed, and transferred onto PVDF membranes. Membranes were blocked with 5% nonfat dry milk in *Tris-buffered saline* with 0.1% *Tween 20* and incubated with goat anti-mouse ICAM-1 (R&D systems), and rabbit anti-β-actin (Cell Signaling Technology) antibodies, followed by incubation with horseradish peroxidase-conjugated secondary antibodies. Signals were detected with an enhanced chemoluminescence solution (Millipore) and quantified by scanning densitometry using a Bio-Image Analysis System (Bio-Rad).

### BBB permeability

Mice were injected with 2% *Evans blue in PBS* (4 ml/kg; Sigma-Aldrich) at 21 hours after ICH induction, followed 3 hours later by transcardiac perfusion. The hemorrhagic brain hemispheres were removed and placed in formamide (Sigma-Aldrich) for 72 hours. The amount of extravasated Evans blue dye was evaluated at 620 nm^49^.

### Neurobehavioral scores

Neurological deficits were assessed by an investigator blinded to the treatment of the animals at 1 and 3 days after ICH. For the quantification of neurological deficits, a 5 point neurological score was employed: 0, no neurological deficit; 1, forelimb weakness; 2, spontaneous circling; 3, partial paralysis on one side; 4, absence of spontaneous movement or unconsciousness; 5, death.

### Statistics

Data are represented as means ± standard errors of the means. Statistical analysis were performed using one-way analysis of variance (ANOVA) followed by Bonferroni’s multiple comparison test. Differences were determined by using the Student two-tailed *t* test when two groups were compared. Behavior data were compared using Mann-Whitney U test. *P* values less than 0.05 were considered as statistically significant.

## Additional Information

**How to cite this article**: Zhu, X. *et al*. von Willebrand factor contributes to poor outcome in a mouse model of intracerebral haemorrhage. *Sci. Rep.* **6**, 35901; doi: 10.1038/srep35901 (2016).

**Publisher’s note:** Springer Nature remains neutral with regard to jurisdictional claims in published maps and institutional affiliations.
